# Quantitative *in vitro *and *in vivo *pharmacological profile of CE-178253, a potent and selective cannabinoid type 1 (CB_1_) Receptor Antagonist

**DOI:** 10.1186/1471-2210-10-9

**Published:** 2010-08-16

**Authors:** John R Hadcock, Philip A Carpino, Philip A Iredale, Robert L Dow, Denise Gautreau, Lucinda Thiede, Dawn Kelly-Sullivan, Jeffrey S Lizano, Xingrong Liu, Jeffrey Van Deusen, Karen M Ward, Rebecca E O'Connor, Shawn C Black, David A Griffith, Dennis O Scott

**Affiliations:** 1 Department of Cardiovascular, Metabolic and Endocrine Diseases, Pfizer Global Research and Development, Groton, CT 06340, USA; 2 Department of Neuroscience, Pfizer Global Research and Development, Groton, CT 06340, USA; 3 Pharmacokinetics, Dynamics, and Metabolism, Pfizer Global Research and Development, Groton, CT 06340, USA; 4 Medicinal Chemistry, Pfizer Global Research and Development, Groton, CT 06340, USA; 5 Roche, 3431 Hillview Avenue S3-2, Palo Alto, CA 94304, USA; 6 Johnson & Johnson Pharmaceutical Research & Development, Welsh & McKean Roads, Spring House, PA 19477-0776, USA

## Abstract

**Background:**

Cannabinoid 1 (CB_1_) receptor antagonists exhibit pharmacological properties favorable for the treatment of obesity and other related metabolic disorders. CE-178253 (1-[7-(2-Chlorophenyl)-8-(4-chlorophenyl)-2-methylpyrazolo[1,5-a]-[1,3,5]triazin-4-yl]-3-ethylaminoazetidine-3-carboxylic acid hydrochloride) is a recently discovered selective centrally-acting CB_1 _receptor antagonist. Despite a large body of knowledge on cannabinoid receptor antagonists little data exist on the quantitative pharmacology of this therapeutic class of drugs. The purpose of the current studies was to evaluate the quantitative pharmacology and concentration/effect relationships of CE-178253 based on unbound plasma concentration and *in vitro *pharmacology data in different *in vivo *preclinical models of FI and energy expenditure.

**Results:**

*In vitro*, CE-178253 exhibits sub-nanomolar potency at human CB_1 _receptors in both binding (K_i _= 0.33 nM) and functional assays (K_i _= 0.07 nM). CE-178253 has low affinity (*K*_i _> 10,000 nM) for human CB_2 _receptors. *In vivo*, CE-178253 exhibits concentration-dependent anorectic activity in both fast-induced re-feeding and spontaneous nocturnal feeding FI models. As measured by indirect calorimetry, CE-178253 acutely stimulates energy expenditure by greater than 30% in rats and shifts substrate oxidation from carbohydrate to fat as indicated by a decrease the respiratory quotient from 0.85 to 0.75. Determination of the concentration-effect relationships and ex vivo receptor occupancy in efficacy models of energy intake and expenditure suggest that a greater than a 2-fold coverage of the K_i _(50-75% receptor occupancy) is required for maximum efficacy. Finally, in two preclinical models of obesity, CE-178253 dose-dependently promotes weight loss in diet-induced obese rats and mice.

**Conclusions:**

We have combined quantitative pharmacology and *ex vivo *CB_1 _receptor occupancy data to assess concentration/effect relationships in food intake, energy expenditure and weight loss studies. Quantitative pharmacology studies provide a strong a foundation for establishing and improving confidence in mechanism as well as aiding in the progression of compounds from preclinical pharmacology to clinical development.

## Background

Cannabinoid receptors are members of the G protein-coupled receptor superfamily [1]. Two cannabinoid receptors, CB_1 _and CB_2_, have been pharmacologically identified. CB_1 _and CB_2 _receptors modulate several downstream signaling pathways including the inhibition of intracellular cyclic AMP accumulation, stimulation of MAP kinase activity and modulation of potassium and calcium channel activities [1]. The fatty acid derivative anandamide was isolated from porcine brain tissue, found to compete for cannabinoid receptor binding and identified as the first endogenous cannabinoid [2]. Other endogenous ligands have been identified, including 2-arachidonylglycerol [3] and archidonylglycerol ether [4]. Anandamide administration leads to a number of pharmacological effects that are similar in nature to THC [5]. As components of the endocannabinoid system have been identified, pharmacological opportunities to modulate the system and effect therapeutic change have been increasingly explored.

The observation that CB_1 _receptor antagonists may be useful as drugs for the management of obesity and metabolic disease was made in 1997 when Aronne and colleagues reported that SR141716A (rimonabant) selectively inhibited sucrose consumption relative to normal chow consumption in male rats [6]. Since this observation, rimonabant has been used extensively in preclinical and clinical settings to define the role of the endocannabinoid system in appetitive (and other) behaviors [7], and more broadly to understand the role of the endocannabinoid system in regulation of energy balance [8-10]. It was hoped that brain-penetrant CB_1 _R antagonists might provide effective therapeutic options for the management of metabolic disorders, such as obesity. Several CB_1 _receptor inverse agonists/antagonists were recently withdrawn from the market or clinical development including the diarylpyrazole rimonabant or SR141716A [11], the acyclic amide taranabant [12], CP-945598 [13], and CE-178253, the focus of the present work.

We previously reported that CE-178253 is efficacious in a model of Parkinsonism [14]. The results suggested that selective cannabinoid CB_1 _antagonism may enhance the antiparkinsonian action of Levodopa and other dopaminomimetics. We herein report the *in vitro *and *in vivo *quantitative pharmacological evaluation of CE-178253, a highly selective and potent CB_1 _receptor antagonist with inverse agonist properties. Furthermore, we demonstrate that CE-178253 is efficacious in preclinical acute and chronic models of FI, energy expenditure and body weight regulation.

## Methods

### Reagents

Human CB_1 _and CB_2 _receptor cDNAs in pcDNA3 (Invitrogen) and/or cell lines were the generous gift of Dr. Debra Kendall (University of Connecticut). The sequences of the receptors were confirmed and are the predominant splice variants. CE-178253 [15], CP-55940 [(1*R*,3*R*,4*R*)-3-[2-hydroxy-4-(1,1-dimethylheptyl)phenyl]-4-(3-hydroxypropyl)cyclohexan-1-ol] were synthesized at Pfizer Global Research and Development, Groton, CT. [^3^H]CP55,940 (158 Ci/mmol) and GTPγ[^35^S] were purchased from Perkin Elmer Life Sciences (Boston, MA). [^3^H]SR141716A (44.0 Ci/mmol) was purchased from Amersham Pharmacia (Piscataway, NJ).

### CB_1 _and CB_2 _receptors and membrane preparations

HEK293 (CB_1_) or CHO (CB_1 _and CB_2_) cells (ATCC) were stably transfected with the human CB_1 _or CB_2 _receptors. Rat brain, and recombinant CB_1 _and CB_2 _and membranes were prepared as described [16]. A Pierce^™ ^BCA kit was used to determine protein concentrations.

### Radioligand Binding Assays

Radioligand binding of CE-178253 to CB_1 _and CB_2 _receptors were performed as described [14]. CP-178253 was diluted in drug buffer (10% DMSO, and 90% TME with 5% BSA,) and then 25 μl was added to each well of a 96-well polypropylene plate. [^3^H]SR141716A was diluted in a ligand buffer (0.5% BSA plus TME) and 25 μl was added to the plate. 10 μg of membranes per well from human CB_1 _and CB _2 _receptor transfected cells and rat brain was used in the assay. The plates were covered and placed in an incubator at 30°C for 60 min. At the end of the incubation period 125 μl of stop buffer (10% BSA plus TME) was added to the reaction plate. The plates were then harvested onto GF/C filter plates (Perkin Elmer Life Sciences) presoaked in BSA (5 mg/ml) plus TME. Each filter was washed twice with TME and dried overnight. In the morning the filters were counted on a Wallac Trilux™ counter.

### GTPγ[^35^S] binding assays at CB_1 _receptors

GTPγ[^35^S] binding assays were performed as described [16]. GTPγ[^35^S] binding assays were performed in a 96-well FlashPlate™ format in duplicate using 100 pM GTPγ[^35^S] and 10 μg membrane per well in assay buffer composed of 50 mM Tris HCl (pH 7.4); 3 mM MgCl2 (pH 7.4); 10 mM MgCl2, 20 mM EGTA, 100 mM NaCl, 30 μM GDP, 0.1% BSA and the following protease inhibitors: 100 μg/ml bacitracin, 100 μg/ml benzamidine, 5 μg/ml aprotinin, and 5 μg/ml leupeptin. The assay mix was incubated with increasing concentrations of antagonist (10^-10 ^M to 10^-5 ^M) for 10 min and challenged with the cannabinoid agonist CP-55940. Assays were performed at 30°C for 1 hr. The FlashPlates™ were then centrifuged at 2000×g for 10 min. Stimulation of GTPγ[^35^S] binding was then quantified using a Wallac Microbeta^® ^[16].

### Receptor Occupancy studies

An *ex vivo *brain receptor occupancy assay was used to calculate the *in vivo *receptor occupancy of CE-178253 at selected doses. The inhibition of specific binding of [^3^H]SR141716A was assessed for CE-178253. Brains were removed from the rats 2 hr after return of food. Brain homogenates were prepared by adding TME buffer to pre-weighed tissue to obtain a working concentration of 50 mg/mL, and then homogenizing with a Polytron for 30 seconds. The homogenate was diluted to a concentration of 2 mg/mL using TME buffer. For the receptor occupancy assay, 160 μL of the diluted brain homogenate was added to the wells of a 96-well polypropylene plate, together with 20 μL of the radioligand [^3^H]SR141716A, (final concentration 2.4 nM; diluted with TME buffer). Triplicate wells were incubated with 20 μL of the cannabinoid agonist CP-55940 (final concentration 10 μM; diluted in TME plus 0.5% BSA and 10% DMSO) to determine non-specific binding. For all other wells, 20 μL of TME was added, and these wells measured total binding. The plates were then covered and incubated for 90 min at room temperature on a plate shaker. Reactions were stopped by the addition of 100 μL ice cold 7.5% BSA in TME. The plates were aspirated then harvested onto GF/C filter plates (Perkin Elmer Life Sciences; Boston, MA) using ice-cold TME buffer. The filter plates had been pre-soaked in 50 μL 0.5% BSA in TME for 60 min. Filters were dried at room temperature for 30 min, after which 25 μL of Microscint™ (Perkin Elmer Life Sciences; Boston, MA) was added to each and the plates analyzed on a Microbeta counter. Care was taken in our assay to minimize drug-tissue dissociation by comparing binding in a time course and under various conditions. The inhibition of specific [^3^H]SR141716A binding was determined by subtracting the proportion of non-specific binding relative to total binding. Experiments were run in triplicate with at least an n = 3 for each treatment group.

### Plasma CE-178253 measurements

Plasma bound fractions for CE-178253 were determined using an equilibrium dialysis assay, as previously described [17]. Spectra-Por 2 membranes (Spectrum Laboratories Inc., Rancho Dominguez, CA) with a molecular cut-off of 12-14 kDa were used for the dialysis. Equilibrium of the system was achieved by incubating the apparatus for 4.5 hr in a 37°C reciprocating water bath (set at 155 rpm). A standard curve was set up over the range 1-1000 ng/mL. All samples were quantified using liquid chromatography/tandem mass spectrometry using a PE Sciex API 3000 spectrometer. For plasma, the unbound fraction was determined as the ratio of concentrations determined in buffer and plasma.

### Food intake assays

All animal studies were approved by the Institutional Animal Care and Use Committee. Male Sprague Dawley rats on normal chow (8 weeks old, 250-300 g on arrival, 300-350 g on test day) were obtained from Charles River. After arrival, animals were individually housed and placed on powdered rat chow. Rats were maintained on a 12-hr light/dark cycle and received food and water *ad libitum*. In all studies CE-178253 and veh (0.5% methyl cellulose) administration to rats and mice was by oral gavage.

For the fast-induced re-feeding assay, food was removed from the cages the afternoon preceding the test day and the rats were fasted overnight. After the overnight fast, rats were administered veh. or CE-178253. Food was reintroduced 30 min after dosing. Food consumption was measured at selected time points as indicated in the figures.

For the spontaneous, nocturnal FI assay, rats were administered veh. (0.5% methyl cellulose) or CE-178253 30 min prior to the start of the dark phase. Food consumption was monitored with electronic scales, and consumption was recorded every 10 min for 12 hr using an automated FI system (Columbus Instruments, Columbus, OH).

### Indirect Calorimetry

Whole body oxygen consumption was measured using an indirect calorimeter (Oxymax from Columbus Instruments, Columbus, OH) in male Sprague Dawley rats. The rats (300-380 g body weight) were placed in the calorimetry chambers and the chambers were placed in activity monitors. All studies were conducted during the light cycle. Prior to the measurement of oxygen consumption, the rats were fed standard chow *ad libitum*. During the measurement of oxygen consumption, food was not provided to the rats. Basal, pre-dose oxygen consumption and ambulatory activity were measured every 10 min for 2.5 to 3 hr. At the end of the basal pre-dosing period, the chambers were opened and the animals were administered a single dose of compound (or veh.) by oral gavage. CE-178253 was prepared in 0.5% methylcellulose as veh. Oxygen consumption and ambulatory activity were measured every 10 min for an additional 1-6 hr after dosing. Resting oxygen consumption, during pre- and post-dosing, was calculated by averaging the 10-min O_2 _consumption values, excluding periods of high ambulatory activity (ambulatory activity count > 100) and excluding the first 5 values of the pre-dose period and the first value from the post-dose period. Change in oxygen consumption is reported as percent and is calculated by dividing the post-dosing resting oxygen consumption by the pre-dose oxygen consumption (X 100). VO_2_, VCO_2_, RER and locomotor activity were all measured.

### Four day rat studies

CE-178253 was evaluated in 4-day chow-fed and DIO S-D rat studies. Male 12-16 week old S-D rats which had been maintained on regular chow were singly housed and acclimated to handling and dosing and two days of food intake were recorded to establish baseline food intake values before dosing was initiated. The animals were randomly sorted and assigned to treatment groups (n = 7-8 per group). Two studies were performed. In study 1 (age = 12 weeks) the mean starting weight of all animals was 370 ± 6 g. In study 2 (age = 16 weeks) the mean starting weight of all animals was 431 ± 11 g. Rats were dosed (2 mls/kg) with veh. or CE-178253 according to body weight. FI and BW were recorded daily.

Male 15 week old S-D rats which had been maintained on a high fat diet (Research Diets, D12079BM, 45% kcal from fat) for 10 weeks were selected for the DIO weight loss study. DIO rats were singly housed were acclimated to handling and dosing and two days of food intake were recorded to establish baseline food intake values before dosing was initiated. The heaviest animals were randomly sorted and assigned to treatment groups (n = 8 per group). The mean starting weight of all animals was 639 ± 8 g. Rats were dosed (2 mls/kg) with veh. or CE-178253 according to body weight. FI and BW were recorded daily.

### DIO Mouse study

Male, 14 week old C57/Bl6/6J mice which had been maintained on a high fat diet (45% kcal from fat) for 6 weeks were selected for the DIO weight loss study. The animals body weights ranged at least 5 standard deviations from age-matched chow-fed control animals mean body weight. Mice were singly housed. The mean starting weight of all animals was 38.9 ± 0.5 g. On Day 0, mice were randomly assigned to treatment groups (n = 10 per group). Mice were dosed daily over 10 days, starting approximately at 30 minutes before the start of the 12 hr dark cycle. BW and food intake were recorded daily.

### Calculations and Statistical analyses

All calculations of *in vitro *receptor characterization were completed using GraphPad Prism™. Statistical analyses of in vivo studies were completed using one-way ANOVA for repeated measures. If the overall result achieved statistical significance (p < .05), then one-way ANOVA was employed for each time point and the results subjected to Fisher's PLSD (least protected significant difference). If the conditions of Fisher's were met, individual two-tailed T-tests compared the treatment group to the veh.-only control group for statistical significance. p < 0.05 was considered statistically significant. Data were excluded from analysis when increased locomotor activity was recorded. All data shown are the mean ± standard error of the mean (SEM) except where noted.

## Results

### *In vitro *pharmacology of CE-178253

#### Radioligand binding

**Figure 1:**
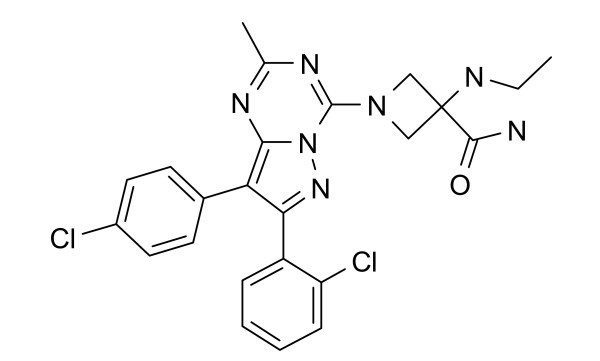
**Structure of CE-178253**.

The structure of CE-178253 is displayed in Figure 1. Saturation and competition radioligand binding assays were used to characterize membranes prepared from human recombinant CB_1 _and CB_2 _and rat brain membranes. [^3^H]SR141716A was used in CB_1 _and rat brain membrane binding assays. [^3^H]CP-55940 was used in CB_2 _binding assays. In membranes prepared from CHO expressing CB_1 _receptors, the K_D _and B_max _of [^3^H]SR141716A were 1.3 nM and 2.3 pmoles/mg, respectively. In membranes prepared from HEK293 expressing CB_1 _receptors, the K_D _and B_max _of [^3^H]SR141716A were 0.9 nM and 1.7 pmoles/mg, respectively. In rat membranes prepared from whole brain the K_D _and B_max _of [^3^H]SR141716A were 2.1 nM and 3.5 pmoles/mg, respectively. In membranes prepared from CHO expressing CB_2 _receptors the K_D _of and B_max _of [^3^H]CP-55940 were 4 nM and 10 pmoles/mg, respectively. The binding affinities were determined using [^3^H]SR141716A, at CB_1 _receptors and rat brain membranes and [^3^H]CP-55940 at CB_2 _receptors. CP-55940 (non-selective cannabinoid receptor agonist) and SR141716A (CB_1 _receptor-selective antagonist) were used in functional assays to characterize the membranes from cells expressing CB_1 _and CB_2 _receptors. Inhibition of CP-55940-stimulated GTPγ[^35^S] binding was used to measure antagonist potency and efficacy at human CB_1 _receptors expressed in CHO cells. CE-178253 exhibits both high affinity binding to and functional antagonism of the human CB_1 _receptor expressed in Chinese hamster ovary (CHO) cells and rat brain cannabinoid receptors (Table 1).

**Table 1:** *In vitro *pharmacological profile of CE-178253

	Competition Binding Assays				GTPγ[^35^S] Functional Assay (human CB_1 _in CHO cells)			
	**K_i _(nM) ± SEM (n)**				**Antagonist**	**Inverse Agonist**		
**Compound**	**Rat brain**	**hCB_1_**	**hCB_2_**	**Selectivity**	**K_i _(nM)**	**IC_50 _(nM)**	**Intrinsic Activity**	**Slope**

CE-178253	0.43 ± 0.13 (5)	0.33 ± 0.07 (5)	15666 ± 6173 (3)	> 30,000	0.07 ± 0.01 (5)	2 ± 0.56 (3)	21%	0.83
SR141716A	0.6 ± 0.1 (32)	1.0 ± 0.1 (73)	285 ± 27 (6)	285	0.54 ± 0.2 (25)	3 ± 1 (2)	30%	1
CP-55940	4.2 ± 0.6 (7)	2.9 ± 1.1 (10)	2.0 ± 0.1 (95)	1.5	Agonist	ND	ND	ND

ND, Not determined

The pharmacological properties of CE-178253, SR141716A and CP-55940 were assessed at human CB_1_, human CB_2 _receptors and rat brain membranes (predominantly CB_1_). The intrinsic activity assessed in inverse agonist assay is the percent decrease in basal GTPγ[^35^S] binding.

The binding affinity (K_i_) of CE-178253 for the human CB_1 _receptor was 0.33 nM (Table 1). Complete inhibition of [^3^H]SR141716A binding was observed at concentrations of CE-178253 greater than 10 nM (not shown). CE-178253 is selective for the human CB_1 _receptor subtype over the human CB_2 _receptor subtype (Ki of CE-178253 > 10,000 nM), as demonstrated by the > 30,000-fold difference in the respective binding K_i _values for these two receptor subtypes.

#### Functional assays

**Figure 2:**
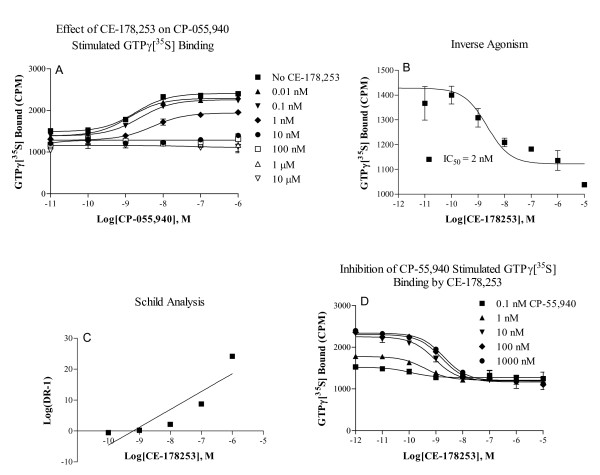
**Functional properties of CE-178253 in GTPγ[^35^S] binding assays**. Panel A. Effect of CE-178253 (0.01 nM-10 μM) on CP-55940 stimulated GTPγ[^35^S] binding. **Panel B. **Inverse agonist assay. **Panel C**. Schild analysis. **Panel D**. Inhibition of CP-55940 stimulated GTPγ[^35^S] binding by CE-178253.

Further in vitro functional profiling demonstrated CE-178253 to exhibit primarily non-competitive CB_1 _receptor antagonist properties and to a lesser degree, competitive antagonism as well. CE-178253 blocked CP-55940-stimulated GTPγ[^35^S] binding in a concentration-dependent manner. CE-178253 (K_i _= 0.07 nM) is almost five-fold more potent in the GTPγ[^35^S] binding functional assay than in the radioligand binding assay (K_i _= 0.33 nM). Schild analysis (Figure 2A-D) in the GTPγ[^35^S] assay confirmed a potency similar to that observed in the binding assays (K_B _= 0.63 nM, slope = 0.83). The intrinsic efficacy and potency of CP-55940 were both decreased by increasing concentrations of CE-178253 (Figure 2A) suggesting that CE-178253 appears to behave as a mixed competitive and non-competitive antagonist. Inverse agonist potency was weaker than functional and binding potencies by 29 and 6-fold, respectively (Figure 2B) [18]. Function of CE-178253 was not assessed at CB_2 _receptors because of the weak potency (K_i _> 10,000 nM) in binding assays.

#### Activity of CE-178253 at other sites

CE-178253 was tested at 1 μM concentration for binding affinity at other receptors, ion channels, and uptake sites (Table 2). CE-178253 did not exhibit any binding activity (as defined by greater than 50% inhibition at 1 μM CE-178253) in these assays. Based on these data CE-178253 is greater than 1000-fold selective over the receptors, enzymes and channels that were tested. Rimonabant exhibits weak agonist activity at the putative third cannabinoid receptor GPR55 [19] with an EC_50 _approximately 500-fold lower than that observed at CB_1 _receptors. No agonist activity was detected with 10 μM CE-178253 at GPR55. Rimonabant also binds weakly to the Vanilloid 1 (TRPV1) receptor channel [20]. TRPV1 was inhibited by 11% (range = 9-13%, n = 2) by 10 μM CE-178253. These data suggest that CE-178253 does not interact with either TRPV1 or GPR55. CE-178253 was not evaluated against TRPM8. Rimonabant has been reported to bind to TRPM8 [21].

**Table 2:** Receptors, ion channels, and uptake sites measured for CE-178253 binding activity

Receptors	Ion Channels/Regulatory sites	Uptake sites
Adenosine (A_1_, A_2a_, A_3_)	Calcium channels:	Choline
Adrenergic (α_1_, α_2_, β_1_, β_2_)	L-type DHP	Dopamine
Angiotensin-II (AT_1_, AT_2_)	L-type (diltiazem)	GABA
Benzodiazepine	L-type (verapamil)	5-HT
Bradykinin (B_1,_B_2_)	N-type	Norepinephrine
Dopamine (D1, D2, D3, D4)		
GABA (non-selective)	**Functional Assays**	
Glutamate (AMPA, kainate, NMDA)	GPR55 (no activity at 10 μM)	
Histamine (H_1_, H_2_, H_3_)	TRPV1 (11% inhibition at 10 μM)	
5-Hydroxytryptamine (5-HT_1A_, 5-HT_2A_, 5-HT_2C_, 5-HT_3_, 5-HT_4_, 5-HT_7_)		
Melanocortin (MC_4_)		
Muscarinic (M_1_, M_2_, M_3_, M_4_)		
Nicotinic (neuronal, muscle)		
Opiate (delta, kappa, mu)		
Platelet activating factor		
Steroid (glucocorticoid)		
Tachykinin (NK_1_)		
Thyroid hormone		
Vasopressin (V_1_, V_2_)		

Inhibition CE-178253 was evaluated for inhibition of binding to the following receptors, channels and sites at 1 μM concentration (except where noted). CE-178253 did not inhibit binding by > 50% at any site noted in the panel.

### *In vivo *pharmacology of CE-178253

As previously reported, CE-178253 dose-dependently reversed the effects of the centrally acting cannabinoid agonist CP-55940 in all four components of the tetrad [14], confirming pharmacological antagonism of central CB_1 _receptor-driven responses.

#### Determination of plasma concentration/effect relationships and brain receptor occupancy of CE-178253 in acute food intake assays

The *in vivo *pharmacology of CE-178253 was evaluated using two models of acute FI: 1) a spontaneous nocturnal feeding paradigm and, 2) an overnight fast-induced refeeding paradigm. Quantitative pharmacology was used to establish concentration/effect relationships based on unbound efficacious plasma concentrations, receptor occupancy and FI efficacy.

**Figure 3:**
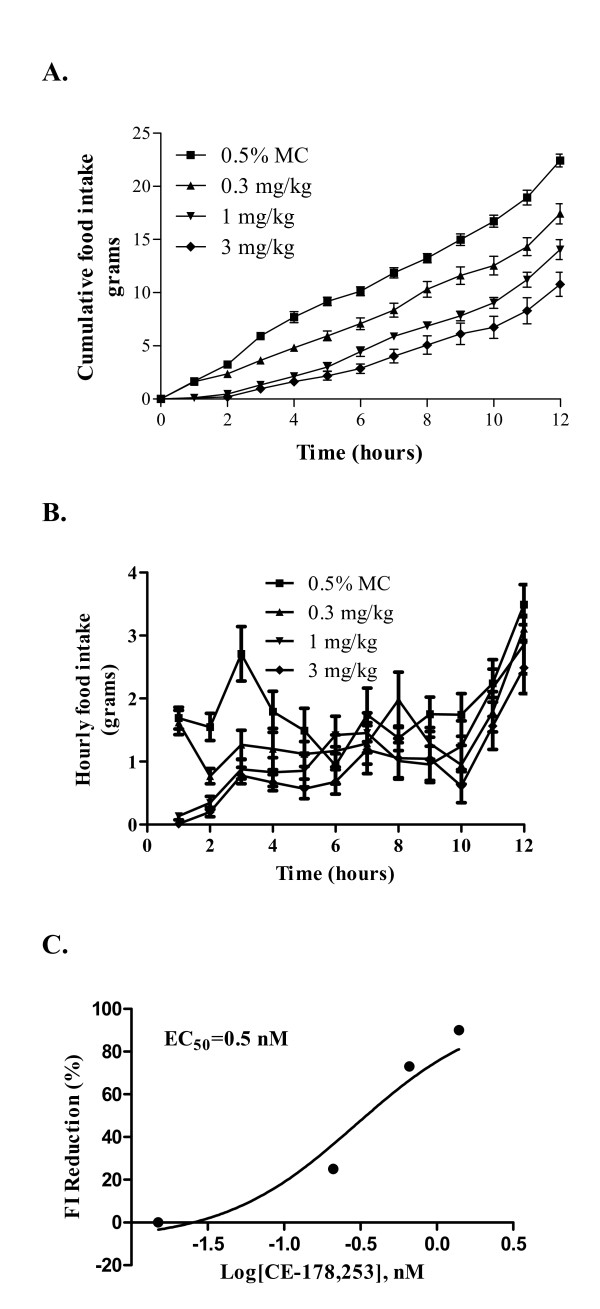
**Effect of CE-178253 in S-D rats on nocturnal phase feeding in spontaneous FI model**. **3A. Time and dose-response relationship of food intake efficacy of CE-178253. **S-D rats were administered veh. (0.5% MC), 0.3, 1 or 3 mg/kg CE-178253 p.o. 30 min prior to the onset of the dark phase feeding cycle. Food was returned 30 min later and cumulative food consumption was measured for 12 hr. Data shown are the Mean +/- SEM, n = 8/group. All time points were statistically significantly different from veh. (p < 0.05) except for the 0.3 mg/kg one hr time point. **3B. Effect of CE-178253 on hourly food intake in S-D rats on nocturnal phase feeding in spontaneous FI model**. S-D rats were administered veh. (0.5% MC), 0.3, 1 or 3 mg/kg CE-178253 p.o. 30 min prior to the onset of the dark phase feeding cycle. Food was returned 30 min later and hourly food consumption was measured for 12 hr. Data shown are the Mean +/- SEM, n = 8/group. All dose groups were statistically significantly different from veh. (p < 0.05) at 2-5 hr time intervals. In addition, the 1 and 3 mg/kg dose groups were statistically significantly different from veh. (p < 0.05) at the 1 and 6 hr time points. **3C. Concentration-effect relationship for CE-178253 in the spontaneous FI model at 2.5 hr post-dose**. S-D rats were administered veh. (0.5% MC), 0.3, 1 or 3 mg/kg CE-178253-01 p.o. 30 min prior to the onset of the dark phase feeding cycle. Food was returned 30 min later and cumulative food consumption was measured for 2 hr. The EC_50 _corresponds to a 50% reduction at 2 hr after the start of the dark cycle feeding phase. Each data point represents the mean of three animals. * = p < 0.05 vs. veh.

The first FI model used for determining anorectic efficacy of CE-178253 was spontaneous nocturnal feeding in rats. In rodents, most feeding activity takes place during the dark phase (nocturnal feeding cycle). In this model, male S-D rats were orally administered compound 30 min prior to the onset of the nocturnal phase. CE-178253 dose-dependently inhibited spontaneous nocturnal FI (0.3 mg/kg, 1 mg/kg and 3 mg/kg, p.o., Figure 3A and 3B) as compared to veh.-treated rats. Cumulative FI was significantly inhibited (p < 0.05) at each hourly time point throughout the dark phase period at each dose tested except at the 0.3 mg/kg dose at the one hr time point (Figure 3A). In addition, the effect of treatment on hourly FI was also assessed. With the exception of the 0.3 mg/kg 0-1 hr interval, CE-178253 (all doses) treatment resulted in a statistically significant decrease in FI through the first four hr compared to veh. No other time points in the interval analysis reach statistical significance. A concentration effect relationship analysis comparing FI reductions to unbound plasma concentrations (C_ave, fu, p, _0-2.5 hr) using the cumulative FI at 2.5 post dose confirmed a concentration-dependent reduction in FI wherein the unbound EC_50 _was calculated to be 0.5 nM (Figure 3B). A 25%, 84% and 94% decrease in FI at the three doses (0.3, 1, 3 mg/kg) compared to veh. at the 2 hr time point was observed. Cumulative food intake at the 2.5 hr time point was 3.2 ± 0.3, 2.4 ± 0.2, 0.5 ± 0.1 and 0.2 ± 0.1 in the veh, 0.3 mg/kg, 1 mg/kg, 3 mg/kg treated rats, respectively.

**Figure 4:**
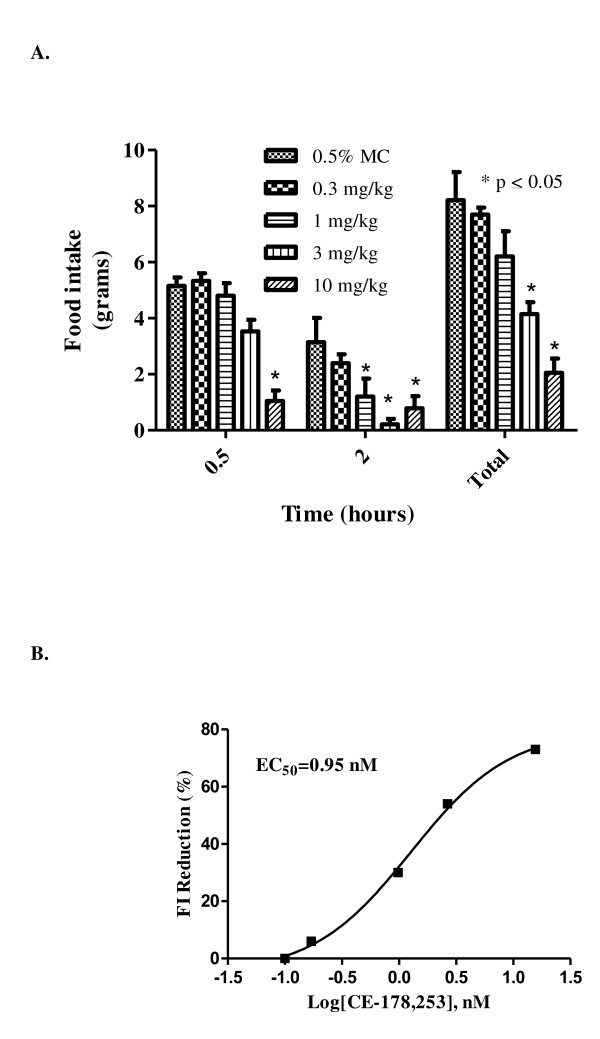
**Effect of CE-178253 in fast-induced refeeding food intake model**. **4A. Dose- and time-dependent effects on cumulative FI in S-D rats by oral administered CE-178253 in the fast-induced refeeding model. **Overnight fasted S-D rats were given veh. (0.5% MC) or 0.3 mg/kg, 1 mg/kg, 3 mg/kg, or 10 mg/kg CE-178253 p.o. in 0.5% MC. Food was returned 30 min after dosing and cumulative food consumption was measured at the 2 hr time point. The number of animals was 8-11 in the veh. and CE-178253 treated groups. Veh.-treated rats ate 8.2 ± 0.3 grams chow. The reduction in FI ± SEM in grams/rat is displayed. * = p < 0.05 vs. veh. **4B. concentration-effect relationship for CE-178253 in the fasted-induced refeeding FI model**. The unbound plasma concentration of CE-178253 was measured at 2.5 hr post-dose. The EC_50 _corresponds to a 35% reduction at two hr post-dose which corresponds to a maximal FI reduction of 70% in this model.

Administration of CE-178253 to male S-D rats inhibited overnight fast-induced refeeding. S-D rats were fasted overnight and veh. (0.5% methylcellulose; MC) or CE-178253 was administered orally. Food was provided to the rats 30 min after dosing. Food intake was measured 30 min and 2 hr after return of food. Dose- and unbound plasma concentration-dependent decreases in cumulative FI versus veh. were observed (Figure 4A, B, Table 3) at both time points after return of food. However, the efficacy (as a percent) was greater in the 0.5-2 hr interval than in the first 0.5 interval. In contrast, FI efficacy in the dark-phase feeding was consistent throughout the first four hr (Figure 3B).

**Table 3:** FI, receptor occupancy, plasma exposure in S-D rats administered CE-178253

Dose (mg/kg, p.o.)	Food Intake % decrease (mean ± SEM)	% Receptor Occupancy (mean ± S-D)	Unbound Plasma Concentration (nM ± S-D )	Unbound plasma/rat brain K_i_
0.3	6 ± 3	27 ± 12	0.17 ± 0.02	0.4
1	25 ± 11	64 ± 2	1.0 ± 0.3	2.3
3	62 ± 5.2*	78 ± 6	2.7 ± 1.0	6.2
10	75 ± 6.2*	82 ± 2	15.6 ± 1.6	36

*significantly different from veh. p < 0.05

Overnight fasted S-D rats were administered veh. (0.5% MC), or CE-178253-01 p.o. Food was returned 30 min later. Cumulative food consumption was measured 2 hr after the return of food.

Brain CB_1 _receptor occupancy by CE-178253, as estimated by *ex vivo *binding, was also dose-dependent (Table 3). The ratios of unbound plasma and total plasma to unbound brain and total brain concentrations were determined at four doses (0.3, 1, 3, 10 mg/kg, p.o.) to assess the brain impairment of CE-178253. This analysis is a useful predictor of the free brain concentration of drugs ([22], [23], [24]). The mean ( ± SD, n = 3 for each dose) of the ratios was 2.9 ± 0.25 suggesting that this compound exhibits, little, if any brain impairment. Rimonabant exhibits a ratio between 1 and 2 (data not shown). These data suggest that a minimum of a 3-fold coverage of the K_i _(calculated 50-75% receptor occupancy and brain/plasma ratios) appears to be required for maximal food-intake reduction.

#### Determination of plasma concentration/effect relationships of CE-178253 in indirect calorimetry studies

**Figure 5:**
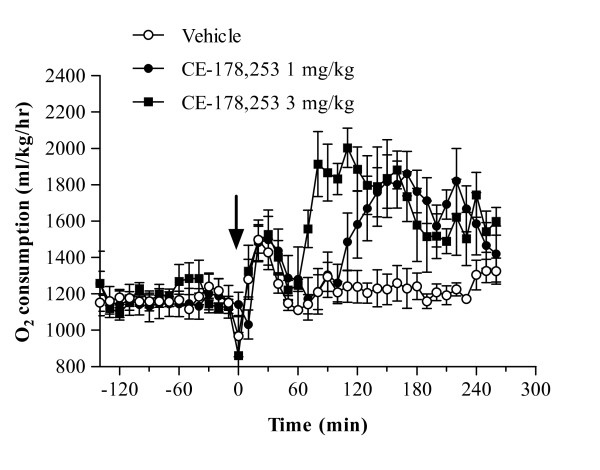
**Stimulation of acute energy expenditure by CE-178253**. Male S-D rats were given veh. (0.5% MC), 1 mg/kg or 3 mg/kg CE-178253 (p.o.). Energy expenditure, measured by indirect calorimetry, was measure for 2 hr before dosing and 4 hr after dosing. The mean ± SEM (n = 8/group) is displayed. * = p < 0.05 vs. veh.

Indirect calorimetry studies measuring oxygen consumption demonstrated that CE-178253 increases energy expenditure (Figure 5). Oral administration of CE-178253 at 1 mg/kg or 3 mg/kg increased average oxygen consumption in S-D rats by 28% and 39%, respectively, between 1 and 3 hr after dosing. No differences in oxygen consumption of veh.-treated or CE-178253 treated rats were observed in the first 1 hr after dosing (Figure 5). These data are consistent with the t_max _of 1 hr that is observed in rats given oral CE-178253 (data not shown). The respiratory quotient (a measure of substrate oxidation) declined from 0.85 to 0.75 over the first hr suggesting a shift from carbohydrate to fat oxidation. A non-statistically significant (p > 0.05) increase in locomotor activity was also observed in all treatment groups. Though the two studies cannot be compared directly the efficacious unbound plasma concentrations (normalized to K_i_) were similar in both FI and indirect calorimetry studies (Tables 3 and 4).

**Table 4:** Oxygen consumption, Respiratory Quotient, and unbound plasma exposure in S-D rats administered CE-178253

Dose (mg/kg, p.o.)	VO_2_, ave, 1-3 hr ( ± S-D)	Respiratory Quotient (VCO_2_/VO2)	Unbound Plasma Concentration (nM ± S-D )	Unbound plasma/rat brain CB_1 _R K_i_
Veh.	3 ± 5	0.85 ± 0.03	NA	NA
1	28 ± 7*	0.74 ± 0.02*	0.8 ± 0.2	1.8
3	39 ± 5*	0.75 ± 0.03*	3.8 ± 0.9	8.8

*significantly different from veh. p < 0.05

In a separate study, core body temperature was measured in C57BL/6J mice. Core body temperature and was found to be increased at the 1 and 3 mg/kg doses by 0.7 ± 0.3 and 1.2 ± 0.3°C (n = 5 per group) compared to veh, respectively.

#### Weight loss efficacy of CE-178253

Chow-fed, lean rats and two animals of obesity, DIO rat and DIO mouse, were used to evaluate body weight changes in response to CE-178253 treatment. A preliminary four day study was performed in chow-fed and DIO rats and followed up with a ten day study in DIO mice.

**Figure 6:**
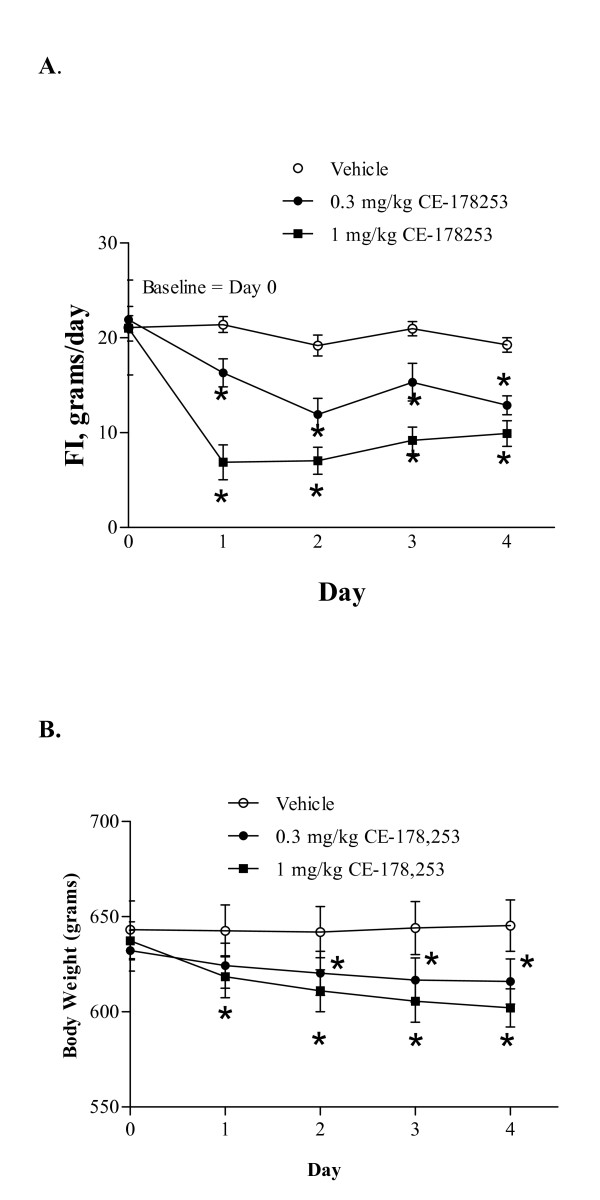
**Effect of CE-178253 on FI (6A) and BW (6B) in DIO rats**. FI and BW were measured in DIO rats (weight = 643 ± 15 g (Veh.), 632 ± 11 (0.3 mg/kg CE-178253) 637 ± 11 (1 mg/kg CE-178253); mean ± SEM, n = 8/group) daily after once daily administration of veh. (0.5% MC) or CE-178253 at either 0.3 mg/kg or 1 mg/kg p.o. FI data shown are the total amount of food consumed expressed in energy grams consumed for each day of treatment. The mean ± SEM (n = 8/group) is shown. * = p < 0.05 vs. veh.

DIO rats were treated once daily with veh. (0.5% MC) or CE-178253 at doses of 0.3 mg/kg p.o. or 1 mg/kg p.o. in a 4-day study (Figures 6A and 6B). CE-178253 dose-dependently reduced daily FI (Figure 6A). The effects on FI were apparent after the first doses and sustained over the 4-day study period. Along with the significant anorectic effect in DIO rats, CE-178253 significantly and dose-dependently reduced body weight over the 4-day study period (Figure 6B). A 3.3% and 5.9% decrease in body weight was observed at the 0.3 and 1 mg/kg doses compared to veh, respectively. In contrast, there was no change in weight in veh-treated rats. Initial and final body weights of veh-treated rats were 643 ± 15 g and 645 ± 14 g, respectively. Initial and final body weights of 0.3 mg/kg CE-178253-treated rats were 632 ± 11 g and 616 ± 12 g, respectively. Initial and final body weights of 1 mg/kg CE-178253-treated rats were 637 ± 11 g and 602 ± 11 g, respectively. A statistically significant difference in BW between veh and the 1 mg/kg dose was observed (p < 0.05).

CE-178253 was also evaluated in chow-fed rats in a 4 day FI and BW study. Rats were treated once daily with veh. (0.5% MC) or CE-178253. In the first study the same doses used in the DIO study were used to compare FI and BW efficacy. In contrast to the 4 day rat DIO study the effects of FI and BW were modest suggesting that CE-178253 is more efficacious in DIO than chow-fed rats (data not shown). CE-178253 was clearly efficacious in acute food intake studies in chow-fed rats up to 12 hours (Figure 3). A comparison of 24 hr FI in chow-fed and DIO rats yielded different efficacy in response to CE-178253 treatment. The 1 mg/kg dose yielded an 8% decrease in 24 hr FI compared to veh-treated animals (p = 0.053). CE-178253 reduced daily FI only on Day 1 in the high dose group whereas significance was observed in the DIO rat study with both low and high doses (Figure 6A). No statistically significant differences in BW changes were observed in the 0.3 and 1 mg/kg dosing groups. Initial and final body weights of veh-treated rats were 371 ± 7 g and 390 ± 7 g (n = 8), respectively. Initial and final body weights of 0.3 mg/kg CE-178253-treated rats were 374 ± 7 g and 388 ± 9 g, respectively. Initial and final body weights of 1 mg/kg CE-178253-treated rats were 373 ± 7 g and 390 ± 9 g, (respectively.

**Figure 7:**
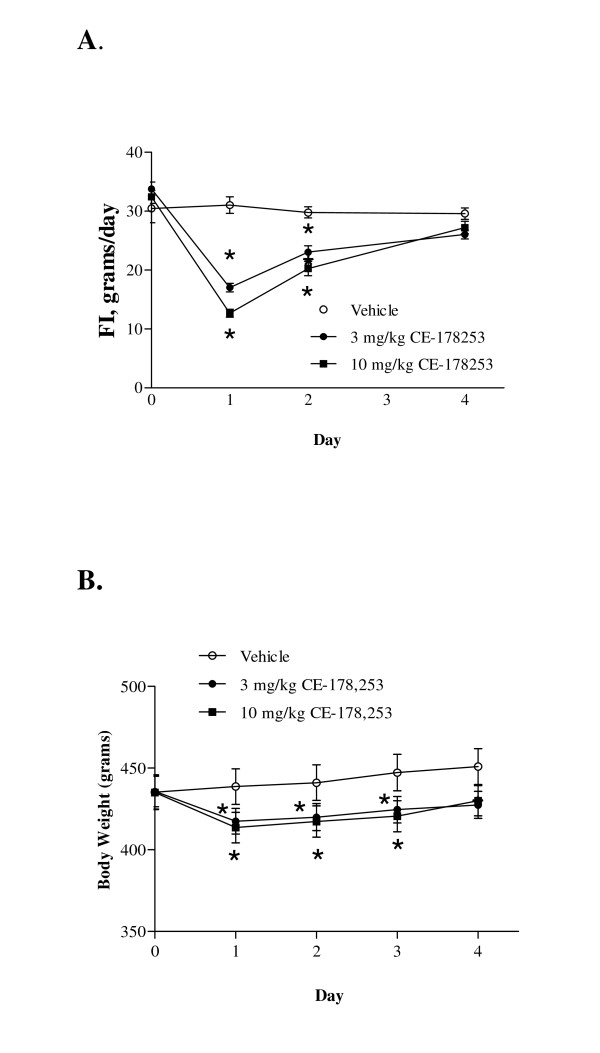
**Effect of CE-178253 on FI (7A) and BW (7B) in chow-fed rats**. FI and BW were measured in chow-fed rats, treated after once daily administration of veh. (0.5% MC) or CE-178253 at either 3 mg/kg or 10 mg/kg p.o. Initial and final body weights of veh-treated rats were 435 ± 11 g and 451 ± 12 g (n = 8), respectively. Initial and final body weights of 3 mg/kg CE-178253-treated rats were 436 ± 9 g and 427 ± 9 g, respectively. Initial and final body weights of 10 mg/kg CE-178253-treated rats were 435 ± 11 g and 430 ± 10 g, respectively. FI data shown are the total amount of food consumed expressed in grams for each day of treatment. The mean ± SEM (n = 6-8/group) is shown. * = p < 0.05 vs. veh.

Based on the weak efficacy observed at the 0.3 and 1 mg/kg doses the study was repeated using higher doses of CE-178253 (Figures 7A and 7B). At 3 and 10 mg/kg (QD, p.o.) doses, statistically significant decreases in FI and BW were observed. Initial and final body weights of veh.-treated rats were 435 ± 11 g and 451 ± 12 g (n = 8), respectively, a 9% gain in BW. Initial and final body weights of 3 mg/kg CE-178253-treated rats were 436 ± 9 g and 427 ± 9 g, respectively, a 2% decline. Initial and final body weights of 10 mg/kg CE-178253-treated rats were 435 ± 11 g and 430 ± 10 g, respectively, 1% increase. As such, the two high dose CE-178253 groups of prevented weight gain that was observed in the veh.-treated rats. These data suggest that efficacy can be maintained but require a higher dose in chow-fed rats. In PK studies, no differences in exposure were observed between chow-fed and DIO rats. Thus, as has been reported for other CB_1 _receptor antagonists it is likely that DIO rats are more sensitive to the anorectic and weight loss effect of CE-178253.

**Figure 8:**
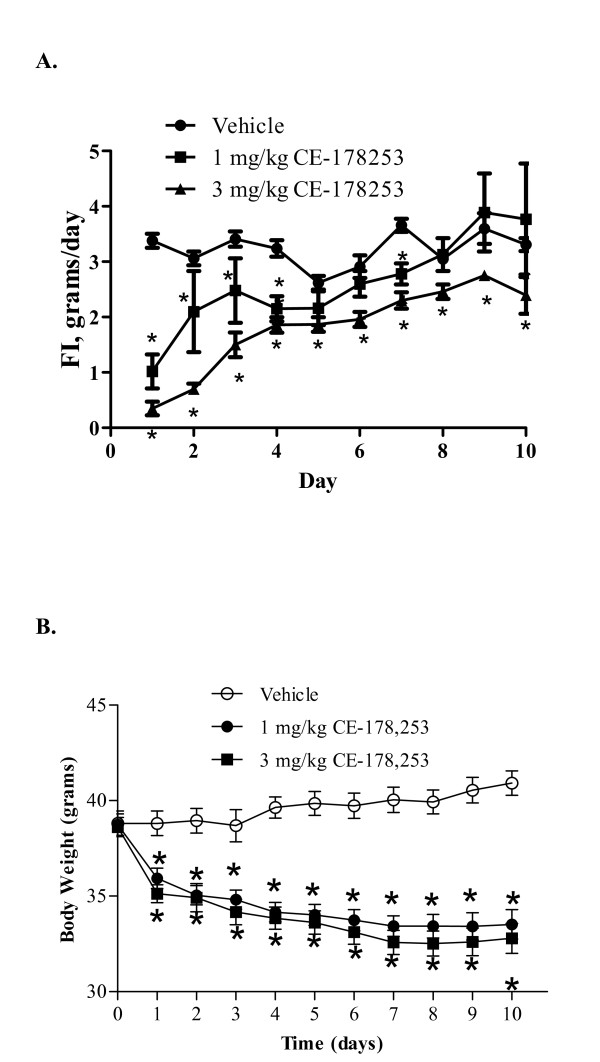
**Effect of CE-178253 on FI (8A) and BW (8B) in DIO mice**. FI and BW were measured in DIO mice (baseline body weight = 38.8 ± 0.7 g (Veh.), 38.7 ± 0.6 (1 mg/kg CE-178253) 38.8 ± 0.6 (3 mg/kg CE-178253); mean ± SEM, n = 10/group) daily after once daily administration of veh. (0.5% MC) or CE-178253 at either 1 mg/kg or 3 mg/kg p.o. FI data shown are the total amount of food consumed expressed in grams consumed for each day of treatment. The mean ± SEM (n = 10/group) is shown. * = p < 0.05 vs. veh.

DIO mice were treated once daily with veh. (0.5% MC) or CE-178253 at 1 mg/kg or 3 mg/kg, p.o., in a 10-day study (Figures 8A and 8B). CE-178253 significantly and dose-dependently reduced FI over the 10-day study. The lower dose of CE-178253 (1 mg/kg, p.o.) provided for a non-significant 8% reduction in FI, whereas the 3 mg/kg dose led to a significant 31% reduction in cumulative FI. As with other CB_1 _receptor antagonists, the food intake reduction was maximal over the first five days and declined over time (Figure 8A). However, FI reductions were still evident on the final day of dosing at the high dose. Along with the anorectic effect in DIO mice, CE-178253 significantly and dose-dependently reduced body weight over the 10-day study period (Figure 8B). The high dose group (3 mg/kg) lost, on average 18% BW.

## Discussion

Endocannabinoids and their receptors are involved in various centrally and peripherally mediated physiological functions including modulation of appetitive behavior, energy metabolism and energy balance [25]. Furthermore, endocannabinoids are elevated in obese humans and rodent models of obesity [26], suggesting a link between an activated endocannabinoid system and obesity. The beneficial effects of pharmacological antagonism of the endocannabinoid system to the treatment of human obesity is well supported by pre-clinical experiments and, more directly, the results of human clinical trials with rimonabant have been shown to be effective in achieving weight loss in long-term studies in humans [27]. In the present study, we have described the *in vitro *and *in vivo *pharmacology of a novel, selective, and orally active mixed competitive and non-competitive CB_1 _cannabinoid receptor antagonist identified as CE-178253.

Cannabinoid receptors selectively couple to the G_i _family of G proteins [1]. CE-178253 displayed a high affinity for CB_1 _receptor binding (0.33 nM), and functional antagonism (0.07 nM) that was highly selective ( > 30,000-fold) compared to CB_2 _receptors. These *in *vitro pharmacological properties, along with the observed lack of activity ( > 50% inhibition of binding at 1 μM CE-178253) at a select panel of receptors, enzymes and ion channels, clearly indicate the desired pharmacological properties of a CB_1 _receptor antagonist as present in CE-178253. SR141716A (rimonabant) was initially considered to be a silent antagonist (i.e., the compound had no intrinsic activity), however studies have reported hyperalgesic, stimulant and immunosuppressive effects of this compound, suggesting that it may be an inverse agonist *in vivo*. In support of these findings, rimonabant has been reported to decrease basal CB_1 _receptor signaling in addition to antagonism of agonist-mediated responses [18]. Our GTPγ[^35^S] binding results with CE-178253 further demonstrate that it is also a weak inverse agonist (relative to the antagonist K_i_) *in vitro *at the CB_1 _receptor. The intrinsic inverse agonist potency of CE-178253 is higher than the efficacious unbound plasma concentration. These data suggest that the observed *in vitro *inverse agonist efficacy of CE-178253 is not critical for efficacy. However, discrimination between inverse agonism endocannabinoid tone *in vivo *is difficult. Apparent inverse agonism *in vivo *may be due to high endocannabinoid tone.

In our pre-clinical assessment of CE-178253, we have shown that the compound is efficacious in two models of FI, fast-induced refeeding and spontaneous, nocturnal FI. Efficacy in both of these models provides evidence that the compound has significant anorectic activity in rodents consistent with its binding affinity. CE-178253 appears to be more efficacious in spontaneous feeding vs. fast-induced refeeding. While robust inhibition of FI efficacy was observed in both models, direct comparison to previous studies is difficult. For example, Kirkham *et al *[28] examined the effect of SR141716A on 2-AG-mediated hyperphagia. The dose of SR141716A used (0.5 mg/kg) in this pharmacological challenge attenuated FI by ~50%. These data are similar to our results in the fast-induced refeeding model where it has been reported that hypothalamic 2-AG levels are high after a fast.

In addition to anorectic efficacy, CB_1 _receptor antagonists also increase energy expenditure (reviewed in 10). A comparison of the quantitative pharmacology between FI and energy expenditure suggest a similar concentration/effect relationship for the two endpoints. The relative contribution, however, of central and peripheral CB_1 _receptors as well as target tissues (muscle or brown adipose) in modulating energy expenditure remains to be elucidated.

Rimonabant [7,29-31] and AM-251 [32], a CB_1 _receptor antagonist structurally similar to rimonabant) treatment decreased body weight in chronic studies in both normal and obese rodents. DIO rodents appear to be more sensitive to the anorectic efficacy of CB_1 _receptor antagonists. Like other CB_1 _receptor antagonists, sustained weight loss was observed with CE-178253 treatment in both DIO rats and DIO mice. In PK studies, no differences in exposure of CE-178253 were observed between chow-fed and DIO rats. However, the FI and BW efficacy the 4 day study in chow-fed rat study required a much higher dose to achieve equivalent efficacy compared to the DIO rats suggesting that CE-178253 is more potent in DIO than chow-fed rats.

## Conclusions

As with all CB_1 _receptors antagonists the relative contributions of FI and energy metabolism to weight loss remains to be determined. None of the above referenced studies provided any quantitative *in vivo *pharmacological analysis. One of the most critical components of increasing confidence in mechanism is the establishment of *in vitro *vs. i*n vivo *concentration effect relationships. The complexities in the understanding of these relationships will vary depending on the stage and available chemical lead matter of a drug discovery program. These can range from concentration/effect relationships in early stage discovery projects to true PK/PD relationships at the later stages of discovery and into development (Scott *et al*, manuscript in preparation). We sought to better understand the concentration/effect relationships of CE-178253 by comparing unbound plasma concentrations, brain receptor occupancy, binding affinity and FI efficacy. Unbound plasma concentrations normalized to K_i _provided a useful benchmark for comparing compounds across different *in vivo *studies and facilitated early predictions of efficacious human plasma concentrations (Scott, DO *et al*, manuscript in preparation). Surprisingly, very little data exist on the *in vivo *quantitative pharmacology of CB_1 _receptor antagonists, a therapeutically important target class. Most studies have relied on dose and not unbound plasma concentrations were not reported. Fong *et al *[33] assessed weight loss efficacy in relation to receptor occupancy. They reported that a minimum of 30% receptor occupancy in a 14 day weight loss study was required to achieve measurable weight loss. It is likely that endogenous cannabinoid levels are high after the fast in the FI studies but not in the indirect calorimetry studies. Thus, a lower concentration of compound could be required for maximal efficacy when assessing energy expenditure compared to FI.

We did not observe changes in FI at this level of receptor occupancy under the standard study conditions. However, CE-178253 reaches steady-state between brain and plasma at a slower rate than other CB_1 _receptors antagonists including rimonabant (data not shown). When the interval between dosing to return of food was extended to 2 hr from 30 min, a statistically significant reduction in FI (27% ± 6, p < 0.05) at 50% receptor occupancy was observed. This efficacy is similar to that observed with other CB_1 _receptor antagonists. For maximal weight loss efficacy, 60-90% receptor occupancy is required for taranabant similar to our observations with CE-178253 receptor occupancy studies.

While brain receptor occupancy studies are in themselves very important, CB_1 _receptor antagonists have pronounced peripheral metabolic effects that appear to be independent of the CNS effects [34]. Having measurements of both brain and unbound plasma concentration provides views of multiple sites of action. These include possible direct effects on adipose, liver, pancreas and muscle, all tissues that are involved in maintaining energy balance [35].

In summary, we have linked *in vitro *(binding and functional), *ex vivo *(brain receptor occupancy) and in vivo (unbound plasma concentrations) to define the quantitative pharmacology and concentration/effect relationships preclinical models of food intake and energy expenditure with the novel CB_1 _receptor antagonist, CE-178253.

## Abbreviations

BSA: bovine serum albumin; CB_1_: cannabinoid receptor type 1; CB_2_: cannabinoid receptor type 2; CHO: Chinese Hamster Ovary; DIO: diet induced obesity; DMSO: dimethyl sulfoxide; EGTA: ethylene glycol tetraacetic acid; GDP: guanosine diphosphate; GTP: guanosine trisphosphate; K_B_: dissociation equilibrium constant for the antagonist; K_i_: dissociation constant for inhibition; p.o.: per os; s.c.: subcutaneously; S-D: Sprague-Dawley; THC: tetrahydrocannabinol; TME: buffer containing TrisHCL, MgCl_2_, and EDTA; C_ave _fu, p: unbound concentration, 0-2 hr, plasma; MC: methyl-cellulose; FI: food intake; veh.: Vehicle, hr.: hour

## Authors' contributions

JRH participated in design of the studies and drafted the manuscript. PAC participated in design and synthesis of CE-178253. PAI participated in design of the studies. RLD participated in design and synthesis of CE-178253, DG participated in design and execution of in vitro pharmacology studies, LT participated in the design and execution of pharmacology studies, DKS participated in design and execution of in vivo pharmacology studies, JSL participated in design and execution of in vitro pharmacology studies, XL participated in design of the studies, JVD conducted LC/MS/MS analysis of plasma samples, KMW participated in the design of pharmacology studies, REO participated in design and execution of in vivo pharmacology studies, SCB participated in design of the studies, DAG participated in design and synthesis of CE-178253, DOS participated in the design of the studies. All authors have read and approve of the final manuscript.
